# Supplementary material for: Molecular signatures of normal pressure hydrocephalus: a large-scale proteomic analysis of cerebrospinal fluid
Source: Fluids Barriers CNS. 2024 Aug 8;21:64. doi: 10.1186/s12987-024-00561-5 (PMC11312837; doi:10.1186/s12987-024-00561-5)

## Supplementary Methods

The proteins that did not pass the Olink batch release quality control criteria were excluded from the study included: KNG1, BMP6, EPHX2, PGLYRP1, EDEM2, CALY, ARL13B, ARNTL, BCL2L11, BID, LTA, GZMB and MGLL, EP300, FGF3, FUOM, KNG1, ADIPOQ, CDHR1, CLSTN1, PSG1, CGA, EFNA1, HTR1B, KCNH2, STXBP1 and YAP1, FLI1, MPI, EBI3_IL27, ANGPTL7, CPLX2, TAGLN3, GABARAPL1, NFKB2, CTAG1A_CTAG1B, OGT, MTHFSD, IFIT1, TNPO1, MAGEA3, SH3GL3, and RAPGEF2.

**Figure s1. Workflow of Intracranial volume (ICV) estimation using FreeSurfer for idiopathic Normal Pressure Hydrocephalus (iNPH) patients.** Patients that had scans with a slice thickness of less than 1 mm (23 out of the 26 iNPH patients) underwent analysis with their original scans (b) . SynthSR, an artificial intelligence (AI) super-resolution tool, was employed to enhance the resolution of the 3 remaining scans, enabling compatibility with FreeSurfer's analysis (a).

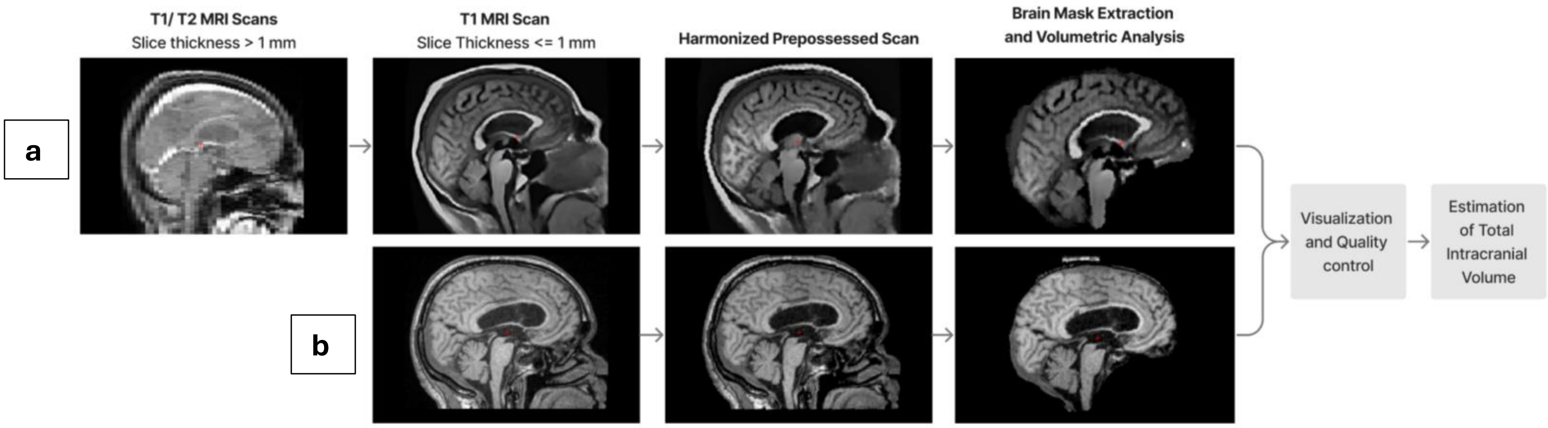

**Figure s2. Boxplots of Analysis of Variance (ANOVA) or of Kruskal-Wallis Analysis of Normalized Protein Expression (NPX) Across Different Conditions.** This visual representation aids in the comparative analysis of protein expression levels (NPX) between conditions such as idiopathic Normal Pressure Hydrocephalus (iNPH), Mild Cognitive Impairment (MCI), and Healthy Control (HC). Each boxplot corresponds to a unique protein demonstrated by their gene symbols, identified through Analysis of Variance (ANOVA) testing across conditions. The significance levels, adjusted using the Benjamini-Hochberg (BH) method, are indicated by asterisks on the plots: p < 0.0001 = '****', p < 0.001= '***', p < 0.01 = '**', p < 0.05 = '*', and 'ns' for non-significant differences.

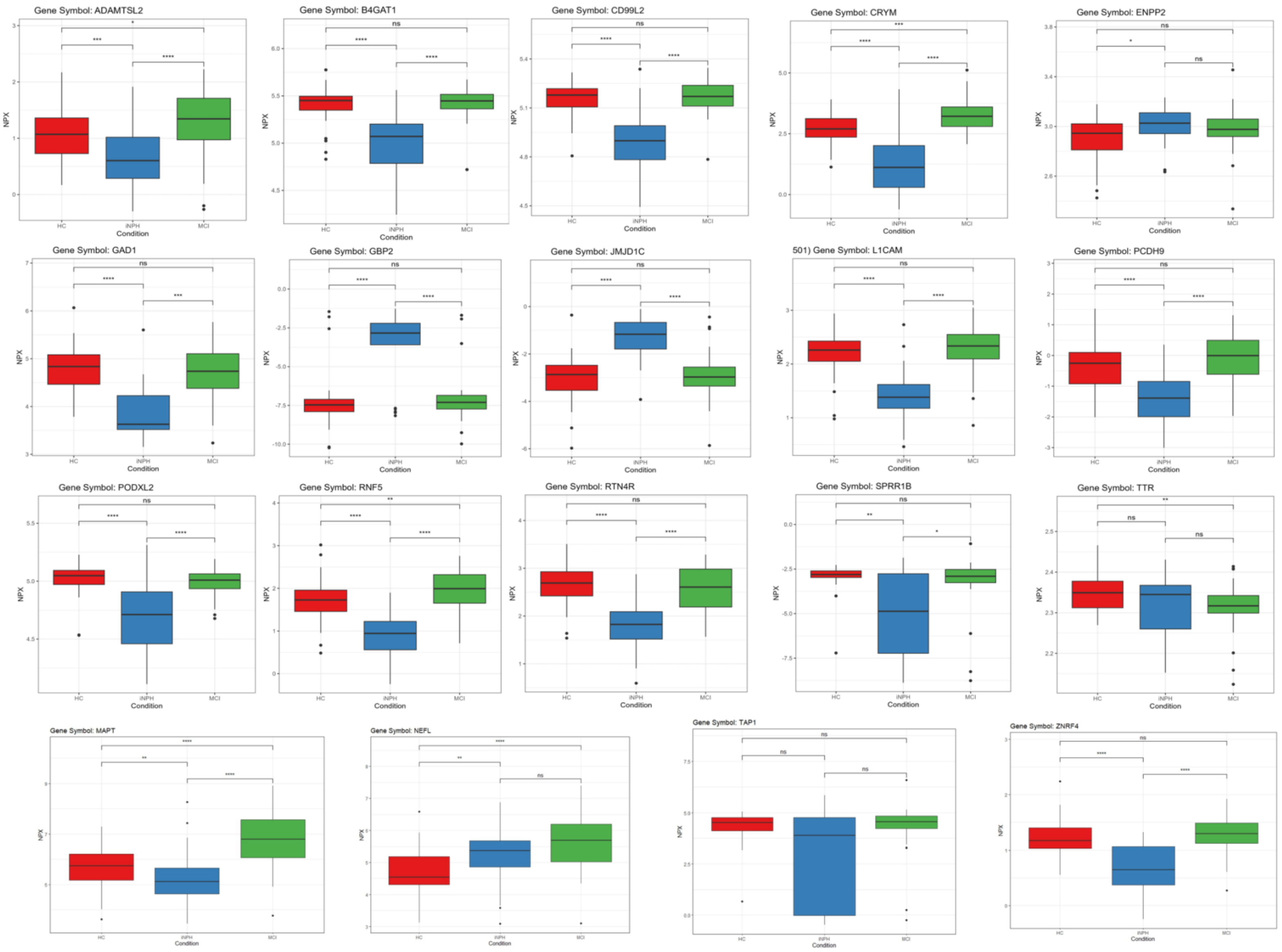

**Figure s3. Over-representation analysis (ORA) results Across Idiopathic Normal Pressure Hydrocephalus (iNPH), Mild Cognitive Impairment (MCI), and Healthy Control (HC) Conditions.** This figure represents the gene ontological (GO) terms of biological processes (BP) between iNPH and HC (a,b) and iNPH and MCI (c, d) that were significant (False discovery rate (FDR) < 0.05).

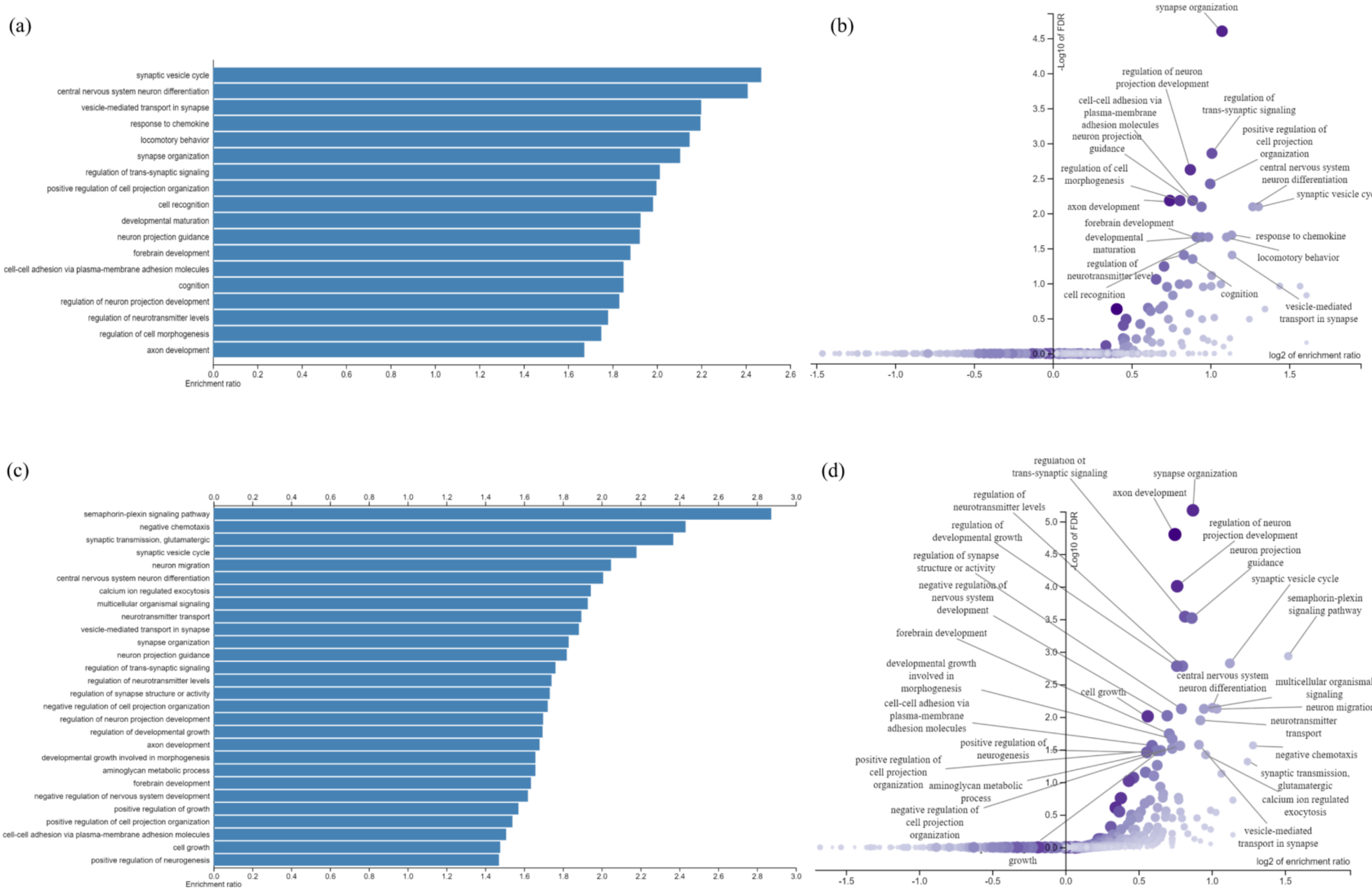

**Figure s4. Principal component analysis (PCA) plot of the of Normalized Protein Expression (NPX) Across Idiopathic Normal Pressure Hydrocephalus (iNPH), Mild Cognitive Impairment (MCI), and Healthy Control (HC) Conditions.** This figure presents a PCA plot based on the NPX data of all proteins, providing a visual representation of the variance and clustering of protein expression profiles across the three studied conditions: iNPH, MCI, and HC. Each data point corresponds to a sample, colored and labeled according to its respective condition.

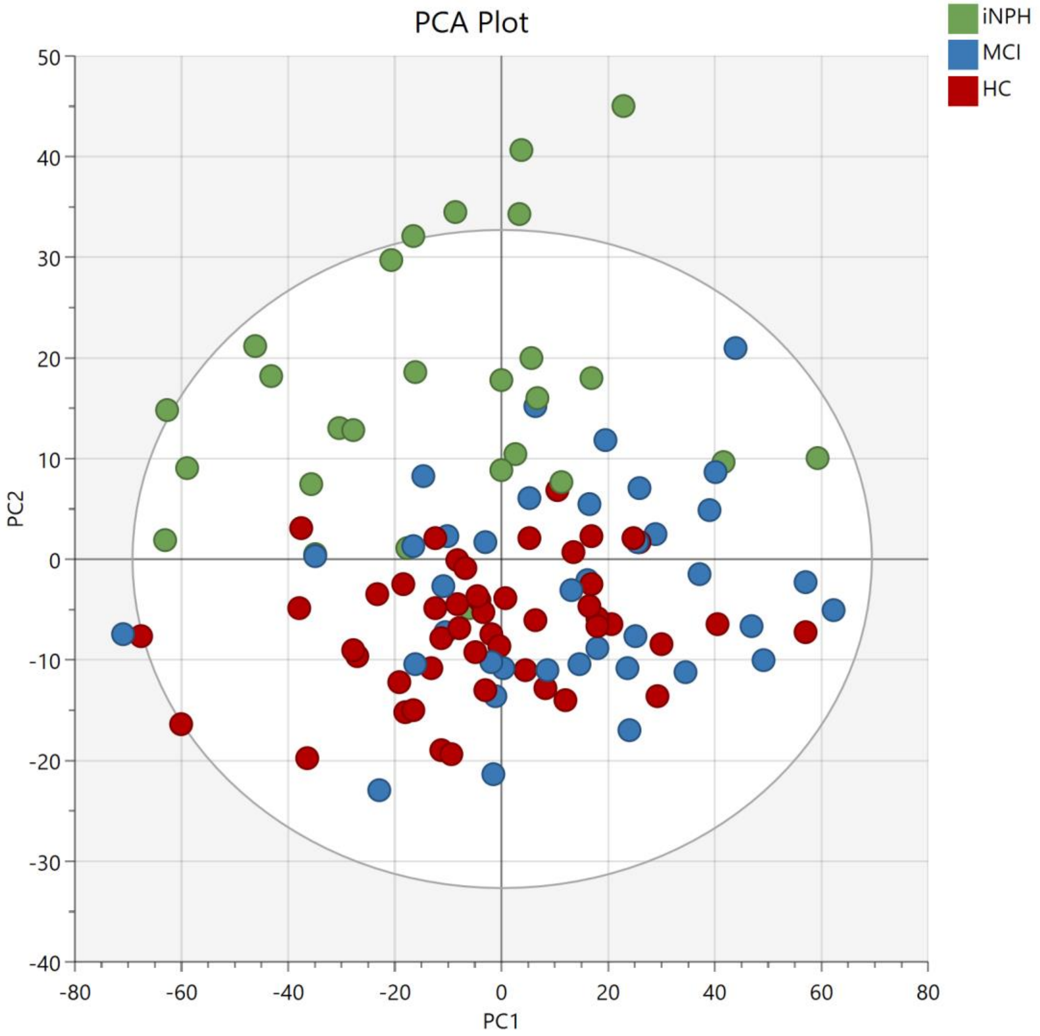

Supplement: Supplementary file 2 — Supplementary Material 2 [file 12987_2024_561_MOESM2_ESM.pdf]
